# Circular data in biology: advice for effectively implementing statistical procedures

**DOI:** 10.1007/s00265-018-2538-y

**Published:** 2018-07-11

**Authors:** Lukas Landler, Graeme D. Ruxton, E. Pascal Malkemper

**Affiliations:** 1 Research Institute of Molecular Pathology (IMP), Vienna Biocenter (VBC), 1030 Vienna, Austria; 2 School of Biology, University of St Andrews, St Andrews, KY16 9TH UK; 3 Department of General Zoology, Faculty of Biology, University of Duisburg-Essen, 45117 Essen, Germany

**Keywords:** Animal navigation, Emlen funnel, Magnetoreception, Biostatistics, Circadian, Chronobiology

## Abstract

Circular data are common in biological studies. The most fundamental question that can be asked of a sample of circular data is whether it suggests that the underlying population is uniformly distributed around the circle, or whether it is concentrated around at least one preferred direction (e.g. a migratory goal or activity phase). We compared the statistical power of five commonly used tests (the Rayleigh test, the V-test, Watson’s test, Kuiper’s test and Rao’s spacing test) across a range of different unimodal scenarios. The V-test showed higher power for symmetrical distributions, Rao’s spacing performed worst for all explored unimodal distributions tested and the remaining three tests showed very similar performance. However, the V-test only applies if the hypothesis is restricted to one (pre-specified) direction of interest. In all other unimodal cases, we recommend using the Rayleigh test. Much less explored is the multimodal case with data concentrated around several directions. We performed power simulations for a variety of multimodal situations, testing the performance of the widely used Rayleigh, Rao’s, Watson, and Kuiper’s tests as well as the more recent Bogdan and Hermans-Rasson tests. Our analyses of alternative statistical methods show that the commonly used tests lack statistical power in many of multimodal cases. Transformation of the raw data (e.g. doubling the angles) can overcome some of the issues, but only in the case of perfect *f-*fold symmetry. However, the Hermans-Rasson method, which is not yet implemented in any software package, outcompetes the alternative tests (often by substantial margins) in most of the multimodal situations explored. We recommend the wider uptake of the powerful but hitherto neglected Hermans-Rasson method. In summary, we provide guidance for biologists helping them to make decisions when testing circular data for single or multiple departures from uniformity.

**Electronic supplementary material:**

The online version of this article (10.1007/s00265-018-2538-y) contains supplementary material, which is available to authorized users.

## Introduction

Circular data are characterized by an inherent periodicity absent from measurements made on a linear scale (such as mass or length). Such data are generated by a range of common measurements across scientific disciplines. Common data are angles (e.g. compass bearings) or measurements over time where an inherent periodicity is relevant: e.g. time of day, seasonality and point in the lunar cycle. Circular data need special treatment in data analysis: consider that an angle of 355° is much nearer to an angle of 5° than it is to an angle of 330°, and so a simple arithmetic mean for example can be quite misleading. Many texts have been dedicated to describing statistical treatment of circular data: e.g. Batschelet (1981), Fisher (1995), Jammalamadaka and SenGupta (2001), Ley and Verdebout (2017), Mardia and Jupp (2000), Pewsey et al. (2013), and some commonly used general statistics texts aimed at biologists also cover this (e.g. Zar 2013). However, there is a lack of clear guidance on how to most effectively test for departure from circular uniformity.

### Unimodal departure from uniformity

The most common statistical exploration of circular data involves testing to see if there is a unimodal bias in the distribution around the circle (i.e. concentration of the data in a certain region of the circle) or whether the null hypothesis that the underlying population involves a uniform spread around the circle is supported. For example, one might test whether the bearings of the initial flights of released homing pigeons are random (uniform) or show a bias towards the direction of the home loft of the pigeons. In this section, we focus on this situation where, if there is departure from circular uniformity, we expect that departure to be unimodal with increased concentration in a single region of the circle (in contrast to multimodal departures such as axial distributions, see next section). By far, the most common statistical test applied to test for departure from uniformity is the Rayleigh test (introduced by Lord Rayleigh in 1880, but also defined in the four monographs listed above). The test statistic for this test is derived from the ‘mean resultant length’ (*r*), which is a measure of the concentration of data points around a circle. The test supports rejection of uniformity for high values of this statistic. Its theoretical foundation lies in the von Mises distribution (e.g. Watson and Williams 1956). The Rayleigh test is known to be the most powerful invariant test against a von Mises alternative to uniformity (Watson and Williams 1956), but its performance against other plausible unimodal alternatives, such as a skew normal or wrapped Cauchy distribution, remains underexplored. This is a significant gap in our knowledge since although the von Mises distribution is often described as the circular analogue of the normal distribution, it does not have a similar mechanistic basis (analogous to the central limit theorem). Thus, there is no theoretical reason to expect von Mises distributions to be commonplace in real datasets. The Rayleigh test is a parametric test, and its original derivation involved the assumption that if there was a deviation from uniformity, that deviation would be of the von Mises form. The robustness of the performance of the test to violations of this assumption are under-explored but will be illuminated further in our study. As well as this essentially parametric test, there is also a range of non-parametric, so-called omnibus tests that make no assumptions about the nature of possible deviations from uniformity and might be used for unimodal departures (see Batschelet 1981 for an overview), but the relative performance of these to each other and to the Rayleigh test is also underexplored (see Stephens 1969). Here, we present simulation analyses that close this knowledge gap for unimodal distributions.

It is important to keep in mind that the tests discussed so far make no assumption about where on the circle any concentration of data might lie if the underlying distribution is non-uniform. In some situations (such as the case of the homing pigeons mentioned above), the researcher may have *a priori* expectation of where any concentration might occur. For these cases, there is a variant of the Rayleigh test (often called the V-test; due to Durand and Greenwood 1958) that allows the mean of the alternative distribution to be specified prior to application of the test. Recently, Ruxton (2017) recommended increased use of this test in large part because it ought to offer increased power over the Rayleigh test. However, again, the quantitative performance of this test relative to the alternatives mentioned above remains unclear. As we will discuss later, the alternative hypothesis for the V-test is identical to a Rayleigh test, and statistical significance alone does not indicate orientation in the *a priori* direction. Interpretation of this test needs care, but evaluation of a confidence interval around the estimated population mean direction can help.

### Multimodal departure from uniformity

In most cases, researchers might be interested in a unimodal departure from uniformity; however, there are many examples where we expect more than one cluster around the circle. For example, we might have data on the times of activity in domestic cats. We might test the null hypothesis that the activity is uniformly distributed throughout the 24 h/day. That is, that the rate of activity is constant and shows no daily variation. From your general knowledge of cats, you might expect that this null hypothesis will be false since cats are crepuscular animals, active around the times of sunrise and sunset. Indeed, what you might expect to see is that there is a peak of activity in the early morning and another peak in the evening. Therefore, you might predict that the data will show a multimodal distribution. As discussed above, the Rayleigh test is known to be the most powerful invariant test against a von Mises alternative to uniformity (Watson and Williams 1956). However, it is well-known that the Rayleigh test (which was designed to detect unimodal von Mises departures from uniformity) has very low power to detect multimodal departures from uniformity (see Batschelet 1981), that is situations with more than one concentration of data around the circle. As argued above, we might expect our imaginary cat-activity data to feature two peaks. The unimodal limitation to the Rayleigh test can be overcome, if we expect that the alternative hypothesis has *f*-fold symmetry for some *f* that can be specified, i.e. multiple peaks are of the same size, symmetrical and evenly spaced around the circle are expected. In this special case, multiplying the raw angles by *f*, and taking their modulus with respect to the maximum allowable angle (2*π* in radians, 360° in angles), will convert *f*-fold symmetric data into a unimodal distribution. However, the requirement for *f*-fold symmetry is restrictive. In the case of our cat-activity example, our two activity peaks are in most situations not expected to be 12 h apart.

As for the unimodal case discussed above, there is a wide range of omnibus tests of circular uniformity that can be used for multimodal deviations from uniformity without the assumption of a von Mises distribution. The three most commonly used of these are Watson’s test, Kuiper’s test and Rao’s spacing test (see Batschelet 1981 for an overview), but the relative performance of these tests against multimodal departures from uniformity has been little explored. The investigation that has been undertaken (Stephens 1969; Bergin 1991; Bogdan et al. 2002; Pycke 2010) has highlighted potential for low power (as discussed above). For this reason, alternative tests have been proposed that were designed specifically to have better power against multimodal alternatives: the two most developed that we could find in the literature were due to Hermans and Rasson (1985) and Bogdan et al. (2002); see Pycke (2010) for theoretical underpinning of general classes to tests for circular uniformity. Both have been subject to very little published evaluation, and neither is available in any software package that we know of. Our aim in this section of the paper is to evaluate these two methods alongside the four more established methods (Rayleigh, Kuiper’s, Watson’s and Rao’s spacing tests) for a range of multimodal distributions. This investigation should offer researchers clear advice for best practice in the common case where circular data have been gathered and there is a desire to explore evidence for departure from uniformity in situations where knowledge of the system gives reason to suspect that such departures might be multimodal. Such advice is urgently required given the multiplicity of alternative tests and given that the limited existing published explorations warn of potential for very low statistical power in some cases (e.g. Stephens 1969; Bogdan et al. 2002).

### Overview

Considering the wide use of circular data and statistics not only in animal behaviour, ecology and behavioural ecology (Wiltschko and Wiltschko 1972; Shimatani et al. 2012), but also in branches of science that range from neurobiology (Taube 2007), chronobiology (Gustafson and Partch 2014), atmospheric sciences (Gaumond et al. 2014) to astrophysics (Archibald et al. 2015), it is concerning that the essential power comparisons are still missing. Our aim in this paper, therefore, is to test the performance of all these alternative tests by simulation over a broad range of different sampling situations. We then use these results to offer researchers clear guidance on how to select the optimal test for the most commonly encountered situations in circular statistics.

## Methods

All simulations were performed in R (R core team 2013), and our code is provided as an electronic supplement (Online Resources 4–7). Graphs were prepared in Prism (GraphPad).

### Power analyses of tests for unimodal distributions

We considered three different unimodal circular distributions: von Mises, wrapped Cauchy and skew normal. The first two are symmetrical distributions fully characterized by two parameters corresponding to the mean value, and a measure of the concentration around that mean. For both distributions, we used a mean of zero (360°), and a range of different values for the concentration parameters (see Chapter 4 of Pewsey et al. (2013) for more on the properties of these distributions). For specified values of the sample size and concentration parameter, we drew samples from these distributions using functions from the ‘circular’ package in R (Agostinelli and Lund 2013). We also considered an asymmetric distribution: the skew normal distribution—the properties of this are described in Azzalini (1985) and Pewsey (2000). This distribution is controlled by the values of three parameters (a location parameter (*ε*) set to zero (360°) throughout, a shape parameter (*α*) set to 30 to give a right skew and a (positive) dispersion parameter (*ω*). For specified sample size, distribution and parameter values (‘model’ was set to null), we generated samples using the rcircmix function from the R package ‘NPCirc’ (Oliveira Pérez et al. 2014). Statistical power to reject the null hypothesis of a uniform distribution is defined as the fraction of 10,000 samples of a given size drawn from a given distribution generating a *p* value less than 0.05. This is by no means a thorough exploration of theoretical distributions for circular data (see Chapter 4 of Pewsey et al. (2013) for an overview of the diversity of these). However, we consider that these three distributions between them capture key features of many empirical unimodal distributions. The von Mises distribution is symmetric about its central tendency and shows a similar ‘bell’ shape to the Normal distribution; the wrapped Cauchy is similarly unimodal and symmetric but generally has the potential to offer a sharper distribution with more concentration near the central tendency than the von Mises. Just as with linear data, there are many circular processes that are not symmetric about the central tendency, and our final distribution, the skew normal distribution, allows exploration of such resulting asymmetric distributions.

We considered a range of different tests, the Rayleigh test, the V-test and three omnibus tests: Watson’s test, Kuiper’s test, and Rao’s spacing test. For the V-test, we included three different scenarios: one where the predicted mean direction coincides with the actual mean of the distribution, one where it is off by 20° and one where the means differ by 45°. All these tests are defined, and their properties discussed in standard textbooks on circular statistics (e.g. Batschelet 1981; Fisher 1995; Mardia and Jupp 2000; Pewsey et al. 2013). These tests are also all available as functions in the circular package in R.

### Power analyses of tests for multimodal distributions

We analysed the power of six different statistical tests for multimodal distributions: Rayleigh test, Watson test, Rao’s spacing test, Kuiper test and two recently proposed Hermans-Rasson and Bogdan tests. The first four tests were calculated using the R library circular (Agostinelli and Lund 2013), using the functions rayleigh.test, watson.test, rao.spacing.test and kuiper.test, respectively. The latter two tests were calculated using our own functions, based on descriptions in the original papers (see Online Resources 2, 3). We applied the Rayleigh test both to the raw data and to data manipulated as described in the introduction to deal with symmetric multimodal situations (multiplying the raw angles by *f*). We conducted power analyses by drawing 10,000 samples from the distribution of interest, performing the statistical test on each of the drawn samples and calculating the proportion of significant test results. We set the alpha levels for all analyses to *p* = 0.05.

In our first analysis set, multimodal von Mises distributions were generated using the rcircmix function from the R library NPCirc (Oliveira Pérez et al. 2014).

We generated a wide variety of multimodal distributions (from two modes up to six modes). For each number of modes, we used two different clustering situations: symmetrically distributed modes (e.g. four modes at 360°, 90°, 180° and 270°) or asymmetrically distributed modes (e.g. four modes at 360°, 60°, 120° and 180°). We then simulated the distributions over the concentration parameter *κ* in 0.1 increments ranging from 0 to 6. This parameter controls the concentration of points around each mode in the underlying distribution from which samples were drawn.

In a second analysis, we analysed the effects on statistical power of differences between the means (a parameter we call Δ) of two distributions, in the case of bimodal data. For this, we generated samples of bimodal distributions where Δ ranged from 0° (when the two modes are coincident) to 180° (when the two modes are diametrically opposite each other on the circle). This analysis was repeated for a range of sample sizes: 15, 25, 40 and 100.

Next, we examined the effect of unequal proportions of two underlying von Mises distributions that combine to give our underlying bimodal distribution. For this, we generated samples from bimodal distributions (either symmetrical, 0° and 180° or asymmetrical, 360° and 90°) and changed the proportion of data points belonging to one of the peaks ranging from 0.05 to 0.5 (in 0.05 steps). This simulation was performed for *κ* = 3 and 2; our exploration of sensitivity to this parameter (see immediately below) suggests that these two values effectively capture the variety of conditions.

Finally, we tested the effects of differing *κ* values between the two von Mises distributions, in a bimodal situation (this analysis was performed for both symmetrical and asymmetrically distributed modes). The *κ* for the first distribution was fixed at 3, but *κ* for the second distribution was changed in 0.1 steps over a range of *κ* values from 0 to 6 (10,000 iterations each).

### Type 1 error calculations

We complimented our power analyses with type 1 error calculation. Drawing 100,000 samples from uniform distributions (using rcircularuniform function in the circular package) and applying the tests described above, this was done for the eight different sample sizes used (10, 15, 20, 25, 30, 40, 80 and 100). The proportion of significant results was then plotted for each sample size.

## Results

### Performance of tests on uniform distributions, and introduction to other distributions considered

**Fig. 1:**
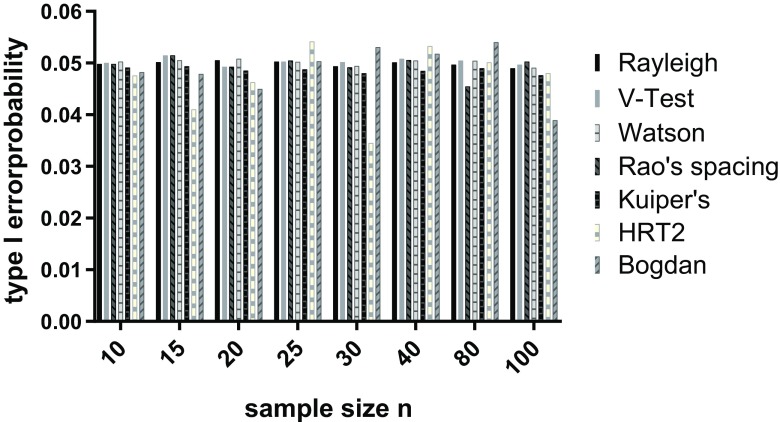
The estimated statistical type 1 error, i.e. proportion of tests generating *p* < 0.05. Estimates are based on 100,000 uniform samples for each of a range of sample sizes: 10, 15, 20, 25, 30, 40, 80 and 100. We compare the Rayleigh test, the three omnibus tests (Kuiper’s, Watson’s and Rao’s spacing tests), the V-test, the Bogdan, and Hermans-Rasson tests

**Fig. 2:**
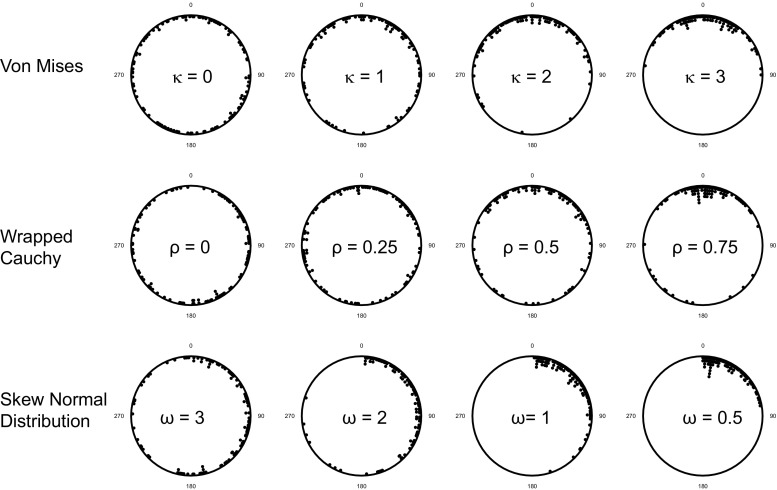
Examples for the distributions used in this analysis. The von Mises distribution, wrapped Cauchy and skewed normal distribution, over a range of concentration/dispersion parameters (values given in the plots, *n* = 100 for each)

When testing the performance of a statistical test, it is important to not only consider the power to detect departures but also the likelihood of falsely detecting departure from uniformity that does not exist, i.e. the type I error rate. We note that all the tests that we consider control type I error rate close to the nominal 5% value (Fig. 1). For some statistical tests, it is important to know the underlying distribution from which the sample has been drawn. For example, the Rayleigh test assumes a von Mises distribution and is most powerful to detect such unimodal departures. To address how the Rayleigh and other tests perform with different underlying distributions, we performed all power analyses with three different distributions: von Mises, skew normal and wrapped Cauchy. Figure 2 shows examples of the three different distributions with varying concentrations around the mean direction.

### Performance of tests on unimodal distributions

**Fig. 3:**
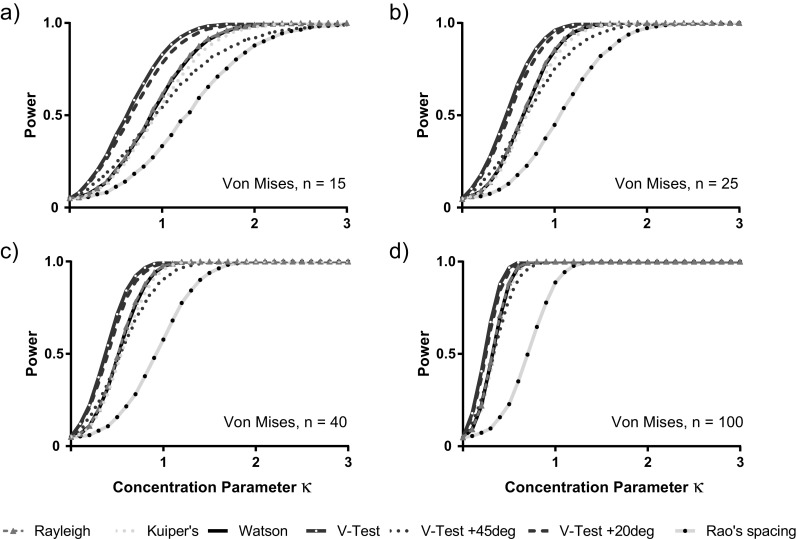
The estimated statistical power to reject the null hypothesis of uniformity based on a sample from a von Mises distribution. Estimates are based on 10,000 samples for each of four sample sizes: **a** 15, **b** 25, **c** 40 and **d** 100. We compare the Rayleigh test, the three omnibus tests (Kuiper’s, Watson’s and Rao’s spacing tests) and three different situations for the V-test (where the test mean value and mean value of the underlying distribution either exactly coincide, differ by 20° or differ by 45°). We obtain estimates for a range of different values of the parameter *κ* that defines the concentration of values for a von Mises distribution

When the sample is drawn from a von Mises distribution, the power to reject the null hypothesis of uniformity increases both with sample size and with how concentrated the underlying distribution is around the central value (how high the concentration parameter *κ* is). We find a consistent pattern among tests in Fig. 3: Rao’s spacing test has the lowest power, and then there is a group of tests of intermediate power (Rayleigh, Watson’s, Kuiper’s and the V-test where the pre-specified test and population mean values differ by 45°), then a group with highest power (V-test with coincident and with 20° different means). Although the V-test with coincident means has higher power than when the means differ by 20°, the effect is slight (less than 5%). The difference in power between these two tests and that of the intermediate group of four tests is strongest when sample size is low and powers are far from either 0.05 or 1, and generally, this difference is of the order of 5–10%. Similar results can be seen for the wrapped Cauchy distribution (Online Resource 1, Fig. A1). There are slight differences when the skew normal distribution is considered (Online Resource 1, Fig. A2). Here, the shape of the graphs flips because the spread of values for this distribution is traditionally described by a dispersion parameter *ω*, such that spread of values increases for increasing *ω* and power decreases accordingly. Interestingly, with the skew normal distribution, there is a change in the relative performance of the different tests. The V-test performs badly in this situation (apart from superior performance at small sample sizes and 2 < *ω* < 3), and Rao’s spacing test again gives relatively poor performance, while there is little difference between the Rayleigh, Kuiper’s and Watson’s tests, which give best performance of all. However, Kuiper’s and Watson’s tests slightly outperform the Rayleigh test in this special situation, especially at high samples sizes, but never offering more than 1–2% more power.

### Performance of tests on multimodal distributions

**Fig. 4:**
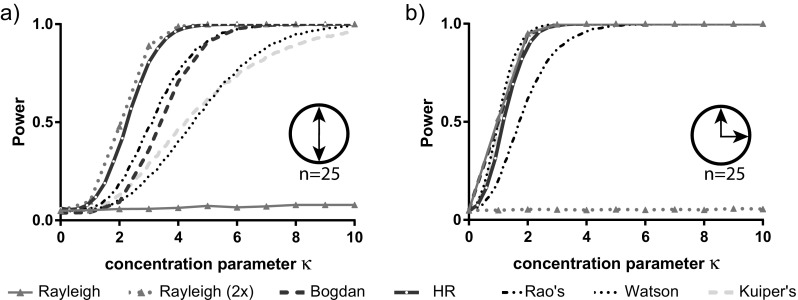
The estimated statistical power to reject the null hypothesis of uniformity based on a sample with two modes each from a von Mises distribution. Estimates are based on 10,000 samples with a sample size of 25. We compare the Rayleigh (2× = modification for two symmetrical modes), Kuiper’s, Watson’s, Rao’s spacing, Bogdan and Hermans-Rasson tests. We obtain estimates for a range of different values of the parameter *κ* that defines the concentration of values for a von Mises distribution. **a** Power estimates for two symmetric modes 180° apart. **b** Power estimates for two asymmetric modes 90° apart

An obvious underlying multimodal population is one with two modes of identical shape and size at opposite ends of the circle (i.e. symmetrically 180° apart). We explore the ability of the different tests to reject the null hypothesis of uniformity from samples of size *N* = 25 from such a distribution in Fig. 4a. We explore performance over a wide range of local shapes of distribution around the two modes, controlled by the concentration parameter (*κ*). The higher the value of *κ*, the more closely data are concentrated about the two modes, and the higher power we have to reject the null hypothesis. It is already known that the Rayleigh test struggles in this situation, and we see this in our simulations. In this case, where the underlying distribution has perfect *f*-fold symmetry (*f* = 2 in our case), then multiplying the measured values by *f* and applying a modulus of the maximum value on the circle (360° or 2*π*) transforms the data into a unimodal distribution. Performing this transformation prior to application of the Rayleigh test then greatly improves the performance of this test. Given that the Rayleigh test is the most efficient test for detecting a von Mises alternative, it is unsurprising that our simulation results show that this ‘Rayleigh (2×)’ procedure offers the best power. However, we note that the performance of the Hermans-Rasson test data is almost as good, without the need to transform the data. The powers of the Bogdan and Rao’s tests are similar, but a little lower than Rayleigh (2×) and Hermans-Rasson. The power curves for the Kuiper and Watson tests are similar and lower again.

It is possible to imagine *f*-fold symmetry to the underlying distribution for any value of *f* (i.e. any number of modes). It is intuitive that the greater the number of modes, the harder the challenge is to detect departure from uniformity on the basis of small samples, and our simulations bear this out. Regardless of the number of modes, the transformation approach prior to application of the Rayleigh test continues to offer the best power, although this power still drops off with increasing number of modes (Online Resource 1; Fig. A3). The only other investigated test that offers valuable power in this situation of perfect *f*-fold symmetry is Rao’s spacing test for *f* = 3. The challenge of this situation is obviously reduced by increasing sample size, but we have observed that power remains low even for large samples if the number of modes is also high (Online Resource 1; Fig. A4).

In Fig. 4b, we break the symmetry of the situation underlying Fig. 4a by moving one of the modes a quarter of the way around the circle, so the two modes are now a quarter of the circle apart in one direction (three quarters in the other direction); otherwise, the situation is unchanged from that of Fig. 4a. We notice that breaking symmetry makes departure from uniformity generally easier to detect. Importantly, breaking the symmetry greatly improves the performance of the Rayleigh test, which now performs very well, but all the explored tests perform well, with only Rao’s spacing test having slightly lower power than the others. These general trends hold for higher numbers of asymmetrically distributed modes (Online Resource 1; Fig. A5).

**Fig. 5:**
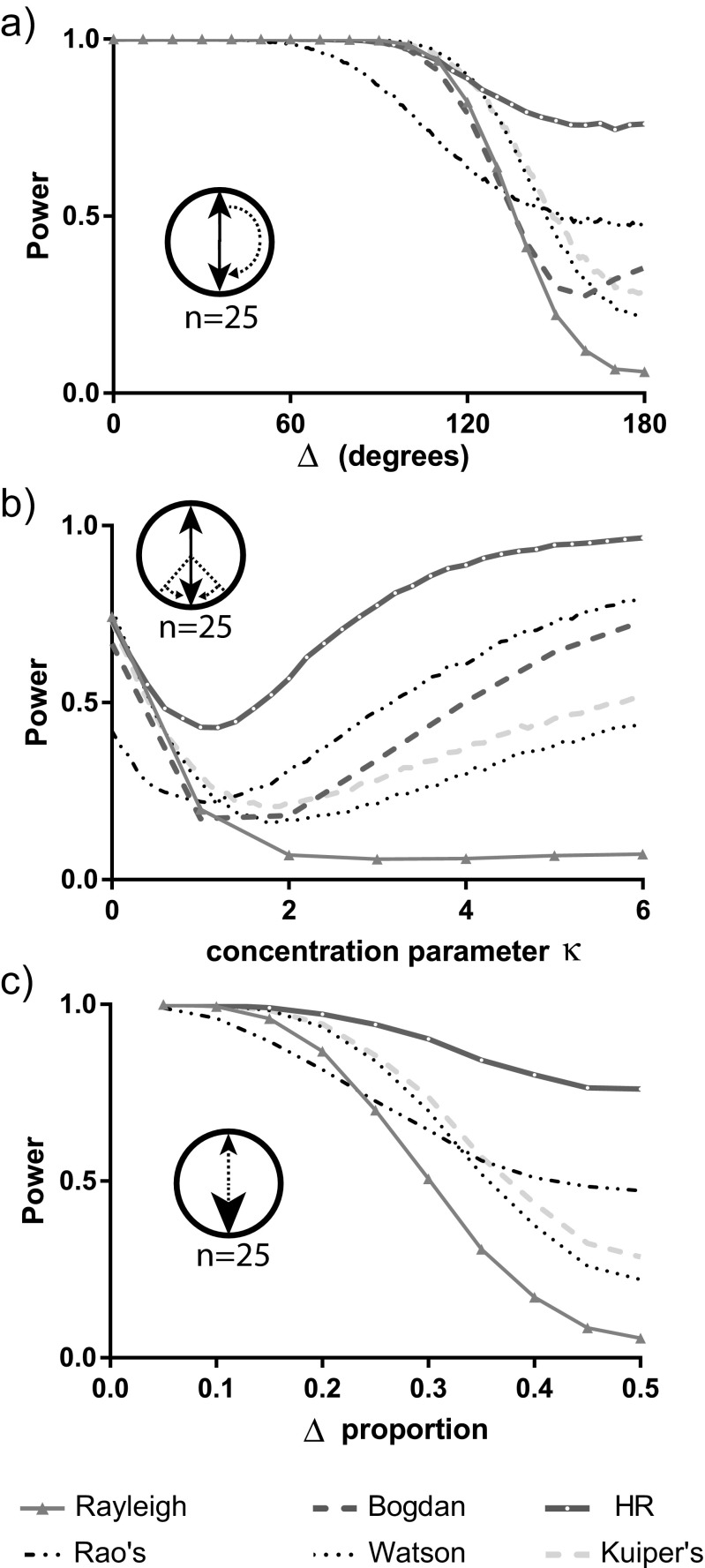
The estimated statistical power to reject the null hypothesis of uniformity based on a sample with two modes each from a von Mises distribution. Estimates are based on 10,000 samples with a sample size of 25. We compare the Rayleigh, Kuiper’s, Watson’s, Rao’s spacing, Bogdan and Hermans-Rasson tests. We obtain estimates for **a** different values of the distance (Δ) between the two modes, **b** different values of the concentration *κ* of one of the modes while holding the concentration of the other mode constant at 3.0, and **c** different weight ratios of the modes (equal weight = a delta proportion of 0.5)

Next, we investigated the relative position on the circle for two otherwise-identical modes. We hold the spread of points around these modes constant and use the *x*-axis to vary the smallest distance between the two modes (Fig. 5a). When this distance is zero, then the modes are coincident (and we have a unimodal underlying distribution), when it is 90°, we have the situation considered in Fig. 4b, and when it is 180°, we have the perfectly symmetric case considered in Fig. 4a. We can see that the Hermans-Rasson method offers the best all-around performance; all other tests show a much more substantial drop in power close to the situation of perfect symmetry. The only other test that shows robustness to this challenge is Rao’s spacing test, but it generally has lower power than the Hermans-Rasson method.

Another way to break the *f*-fold symmetry would be to have different spreads of points about the modes. In Fig. 5b, we have two modes at opposite points on the circle again, and the two models have equal weight (in that the data-points in the sample are just as likely to be associated with one mode as the other), but the spread of data-points about the two modes can differ. We hold the concentration about one mode constant and vary the concentration parameter associated with the other mode over a range of concentrations as described along the *x*-axis. The figure emphasizes several themes already seen: the challenge of detecting perfect *f*-fold symmetry, the poor performance of the Rayleigh test when modes are symmetrically distributed and the broadly good performance of the Herman-Rasson test. If we break symmetry by varying the weights of the two distributions instead of the spread (Fig. 5c), then this greatly improves the performance of the Rayleigh test, but we did not find circumstances where it (or any of the others) consistently substantially outperformed the Hermans-Rasson test.

## Discussion

### Recommendations for unimodal distributions

Based on our simulation results, we can offer very clear guidance on testing for unimodal departure from circular uniformity. Simply put, at present, there seems no compelling evidence to depart from adoption of the commonly used Rayleigh test. Only in a situation where a single a priori specified mean direction is of interest to the researcher can the V-test offer superior power to the more general Rayleigh test. This advantage must be set against the need for care in interpreting a significant departure from uniformity by this test. The V-test on its own does not provide compelling evidence of departure concentrated about the specified direction, and our simulations demonstrate that even departures centred quite far from the specified direction can trigger significant results using this test (Fig. 3). However, if there is only one direction of interest, the V-test is the best choice, especially if sample sizes are low and power becomes an issue. In such cases, there may also be a philosophical reason for adopting the V-test rather than the Rayleigh test, because the alternative hypothesis provides a better match to the fundamental question that is being explored. It is important, however, that a significant test result from a V-test must *always* be interpreted in combination with a confidence interval for the population mean value. The confidence interval can easily be calculated, see Pewsey et al. (2013)—who also provide suitable *R* code. If the confidence interval does not include the pre-specified value, then the researcher should conclude that the data do not support their alternative hypothesis, but if the confidence interval is narrow and includes this value, then there is evidence to support it. Uncertainty arises, however, if the confidence interval includes the pre-specified value but is relatively wide. In this case, the conclusion must be very tentative: The data could be interpreted as being in accord with the alternative hypothesis, but it is also in accord with other explanations. In brief, there is a price to be paid for the extra power offered by the V-test: Having pre-specified one possible position of concentration of the data to gain extra power to test this possibility, the researcher loses the ability to consider any other potential departures from uniformity. In fairness, once the V-test has been applied, researchers must avoid any speculation about support in the data for alternatives to uniformity other than the one they pre-specify in their V-test. We would also emphasize that the mean value to be tested in a V-test should be decided *before* any preliminary inspection of the data; if this value is influenced in any way by inspection of the data and then the V-test is applied to that same data, then the type I error rate will be substantially increased.

Of all the unimodal situations that we explored, we did not find one where any of the commonly used alternative tests offered substantially better performance than the Rayleigh test. The Kuiper’s and Watson’s tests give almost similar power, but Rao’s spacing test offers consistently lower power. Given the relative unfamiliarity of Kuiper’s and Watson’s tests, we see no reason to recommend their use over the Rayleigh test. It is of course possible to further expand the results presented here by studying different sample sizes, or different unimodal distributions, but we do not see strong reason to expect that such explorations will produce results that significantly affect our recommendations above.

### Recommendations for multimodal distributions

Our results emphasize that detecting symmetrically distributed multimodal departures from uniformity when the number of modes is greater than two is a challenge. If researchers expect to be in such a position, then our advice would be to maximize the size of their sample and then apply the Hermans-Rasson test but still expect relatively low power to reject the null hypothesis. Although the ‘trick’ of transforming a multimodal distribution can be effective, it relies on three requirements: (i) The number of modes can be identified correctly (model-fitting approaches might be useful for helping identify this number (e.g. Fitak and Johnsen 2017), (ii) these modes are symmetrically distributed around the circle and (iii) the local distribution around each mode is itself symmetrical. We suspect that these criteria will only be met in special cases in practice, but in these cases, the Rayleigh test offers superior performance. Multimodal departures that are not symmetrically distributed can readily be detected by all tests considered here. Our results allow several conclusions to be drawn about the relative merits of the different multimodal tests. The Rayleigh test is the most commonly used test for departure from circular uniformity, and its performance against unimodal departures is very good. Thus, when researchers suspect (or are only interested in) unimodal departures from uniformity, the Rayleigh test can be recommended, but in the multimodal case, they should seek an alternative offering better performance.

When researchers suspect that departure from uniformity might be multimodal, then we recommend the method of Hermans-Rasson as offering the best performance across a wide range of situations. There is no particular multimodal situation in which any other test performs substantially better than this test. Although the test is not available in any software package, it is relatively simple to code and we offer a full description of the procedure and its implementation in R in Online Resources 2 and 3.

Although the modern method of Bogdan et al. (2002) offers generally improved performance over older methods, it does not perform as well as that of Hermans-Rasson in the situations that we explore and is conceptually and computationally more complex. Hence, we do not strongly recommend its wider uptake—although for completeness, we provide a full description of the procedure and its implementation in R in Online Resources 2 and 3.

For the researcher who would rather avoid these modern methods and use one of the three omnibus methods provided in existing software, it is difficult to offer clear guidance. We found no circumstances where there was substantial difference between Watson’s and Kuiper’s tests. In situations where all tests perform relatively well, Rao’s spacing test tends to have lower power than either Watson’s or Kuiper’s tests, but it is more resistant than the other two to drop in power in situations where testing is difficult. Pending further research, researchers should consider themselves free to select whichever of these three tests is traditionally used in their discipline. However, we hope that the results presented here will encourage the implementation of the Hermans-Rasson test in easy-to-use software packages. Pending such developments, R users may find our implementation of some value.

## Conclusion

We explored the power of a wide range of approaches to test non-uniformity of distributions (see Table 1 for a summary of our recommendations). When researchers expect a unimodal departure and cannot a priori define the expected mean direction, the Rayleigh test should be used. In case there exists a predefined mean direction, the V-test offers most power. For multimodal departures, the Hermans-Rasson test performed very well. However, in cases of symmetrical multiple modes, all tests perform poorly. The most powerful approach in such cases is a transformation of the *f*-fold symmetry into a unimodal situation and applying a Rayleigh test. In this case, the number of symmetrical modes must be defined a priori in order to avoid alpha error inflation. Our paper provides up-to-date and comprehensive comparisons of available tests, with clear guidelines which approach to use in which situation. We note, however, that we have considered only continuously distributed data, and for aggregated (grouped) circular data, we recommend Humphreys and Ruxton (2017) as a useful starting point.

**Table 1:** Summary of our recommendations based on the simulations presented. In all cases, confidence intervals need to be calculated in order to provide evidence for clustering around a defined direction. Expected directions (dir.) must be set a priori; similarly if an *f*-fold transformation is used, it has to be decided on or before data inspection

Expected distribution	Test recommended
von Mises (unimodal)-expected dir.	V-test
Other circular dist. (unimodal)-expected dir.	V-test
von Mises (unimodal)-no expected dir.	Rayleigh test
Other circular dist. (unimodal)-no expected dir.	Rayleigh test
von Mises (bimodal-modes mirrored)	Rayleigh test (after *f*-fold transformation)
von Mises (bimodal-mode-symmetry unknown)	Hermans-Rasson test
von Mises (> 2 modes-modes mirrored)	Rayleigh test (after *f*-fold transformation)
von Mises (> 2 mode-symmetry unknown)	Hermans-Rasson test

## Electronic supplementary material

ESM 1 (PDF 1026 kb)

ESM 2 (PDF 202 kb)

ESM 3 (PDF 109 kb)

ESM 4 (R 2 kb)

ESM 5 (R 1 kb)

ESM 6 (R 2 kb)

ESM 7 (R 2 kb)
