# Supplementary material for: Circular data in biology: advice for effectively implementing statistical procedures
Source: Behav Ecol Sociobiol. 2018 Jul 11;72(8):128. doi: 10.1007/s00265-018-2538-y (PMC6060829; doi:10.1007/s00265-018-2538-y)
Supplement: Supplementary file 1 — (PDF 1026 kb) [file 265_2018_2538_MOESM1_ESM.pdf]

Online Resource 1: Supplementary figures

Behavioural Ecology and Sociobiology

Circular data in biology: Advice for effectively implementing statistical procedures

Lukas Landler[1], Graeme D. Ruxton[2], E. Pascal Malkemper[1,3]

Affiliations

1 Research Institute of Molecular Pathology (IMP), Vienna Biocenter (VBC), Austria

2 School of Biology, University of St Andrews, St Andrews KY16 9TH, UK

3 Department of General Zoology, Faculty of Biology, University of Duisburg-Essen, 45117 Essen, Germany

Corresponding author email address: pascal.malkemper@imp.ac.at

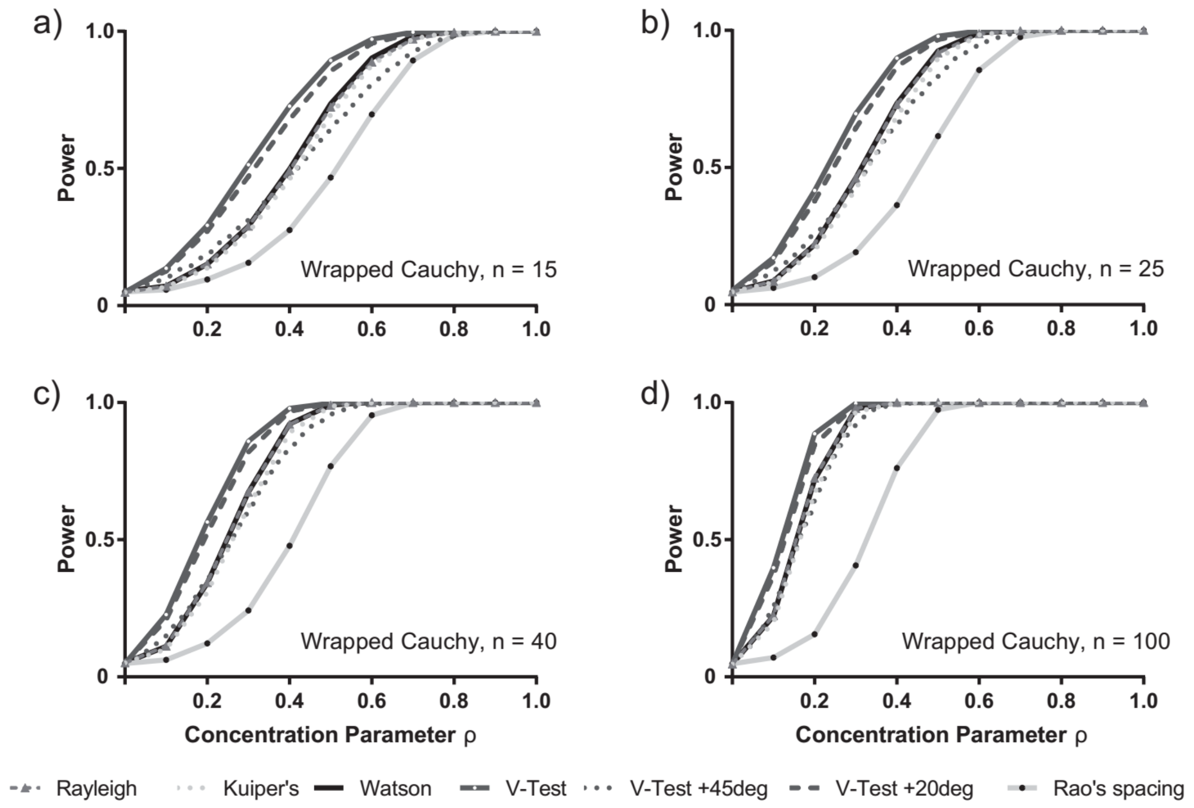

Fig. A1 The estimated statistical power to reject the null hypothesis of uniformity based on a sample from a wrapped Cauchy distribution. Estimates are based on 10 000 samples for each of four sample sizes: 15, 25, 40 and 100. We compare the Rayleigh test, the three omnibus tests (Kuiper's, Watson's and Rao's spacing tests) and three different situations for the V-test (where the test mean value and mean value of the underlying distribution either exactly coincide, differ by 20° or differ by 45°). We obtain estimates for a range of different values of the parameter ρ that defines the concentration of values for a wrapped Cauchy distribution

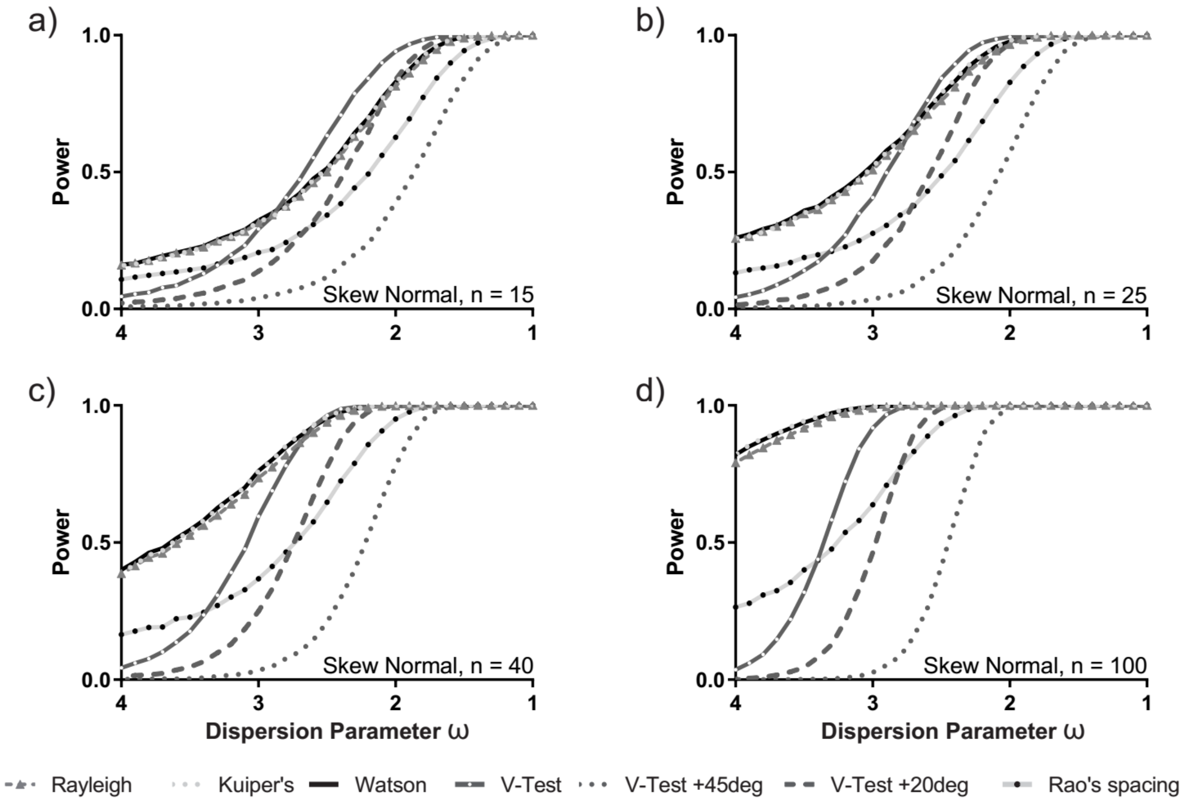

Fig. A2 The estimated statistical power to reject the null hypothesis of uniformity based on a sample from a skew normal distribution. Estimates are based on 10 000 samples for each of four sample sizes: 15, 25, 40 and 100. We compare the Rayleigh test, the three omnibus tests (Kuipers, Watson's and Rao's spacing tests) and three different situations for the V-test (where the test mean value and mean value of the underlying distribution either exactly coincide, differ by 20° or differ by 45°). We obtain estimates for a range of different values of the parameter ω that defines the dispersion of values for a skew normal distribution (higher values = higher dispersion)

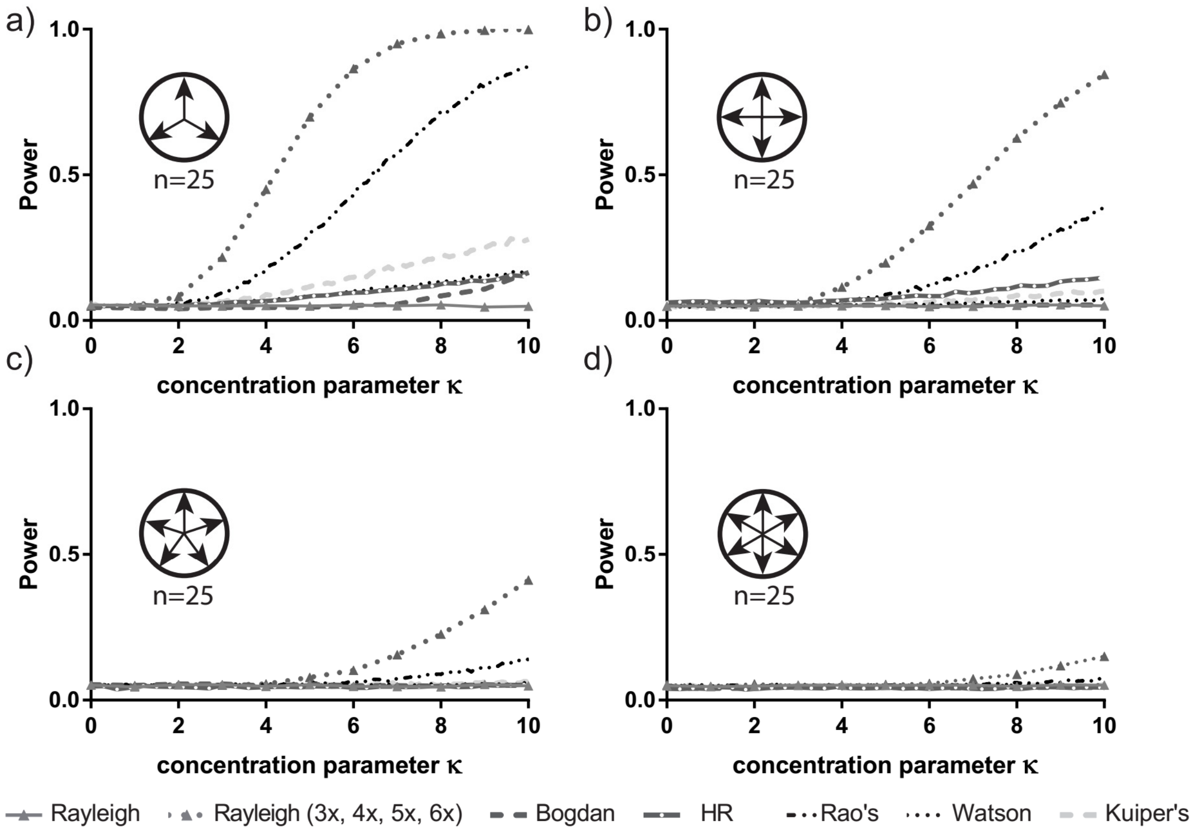

Fig. A3 The estimated statistical power to reject the null hypothesis of uniformity based on a sample with a) three, b) four, c) five, d) six modes distributed symmetrical around the circle. Estimates are based on 10 000 samples with a sample size of 25. We compare the Rayleigh (3x, 4x, 5x, 6x = modifications for symmetrical modes), Kuiper's, Watson's, Rao's spacing, Bogdan and Hermans-Rasson tests. We obtain estimates for a range of different values of the parameter κ that defines the concentration of values for a von Mises distribution

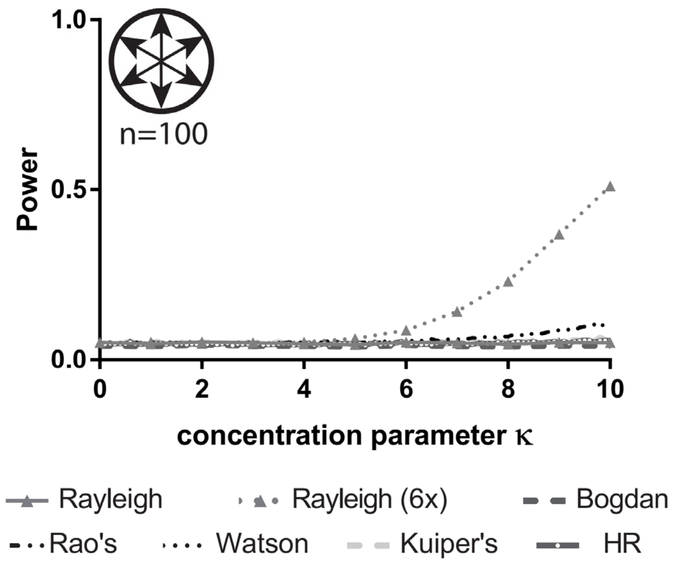

Fig. A4 The estimated statistical power to reject the null hypothesis of uniformity based on a sample with six modes distributed symmetrical around the circle. Estimates are based on 10 000 samples with a sample size of 100. We compare the Rayleigh (6x = modification for six symmetrical modes), Kuiper’s, Watson’s, Rao’s spacing, Bogdan and Hermans-Rasson tests. We obtain estimates for a range of different values of the parameter κ that defines the concentration of values for a von Mises distribution

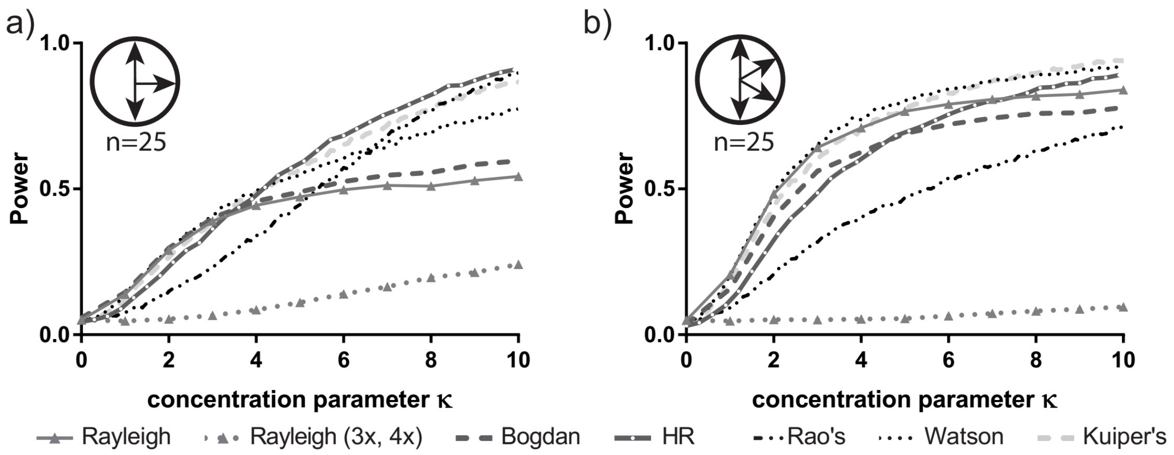

Figure A5: The estimated statistical power to reject the null hypothesis of uniformity based on a sample with a) three, b) four modes distributed asymmetrical around the circle. Estimates are based on 10 000 samples with a sample size of 25. We compare the Rayleigh (3x, 4x = modifications for symmetrical modes), Kuiper's, Watson's, Rao's spacing, Bogdan and Hermans-Rasson tests. We obtain estimates for a range of different values of the parameter κ that defines the concentration of values for a von Mises distribution
