# Supplementary material for: Circular data in biology: advice for effectively implementing statistical procedures
Source: Behav Ecol Sociobiol. 2018 Jul 11;72(8):128. doi: 10.1007/s00265-018-2538-y (PMC6060829; doi:10.1007/s00265-018-2538-y)
Supplement: Supplementary file 2 — (PDF 202 kb) [file 265_2018_2538_MOESM2_ESM.pdf]

Online Resource 2: Description of recently proposed methods for testing the null hypothesis of circular uniformity

Behavioural Ecology and Sociobiology

Circular data in biology: Advice for effectively implementing statistical procedures

Lukas Landler[1], Graeme D. Ruxton[2], E. Pascal Malkemper[1,3]

Affiliations

1 Research Institute of Molecular Pathology (IMP), Vienna Biocenter (VBC), Austria

2 School of Biology, University of St Andrews, St Andrews KY16 9TH, UK

3 Department of General Zoology, Faculty of Biology, University of Duisburg-Essen, 45117 Essen, Germany

Corresponding author email address: pascal.malkemper@imp.ac.at

The method of **Bogdan, Bogdan & Futschik (2002):**
We have a set of *n* measurements $\alpha_1,\ldots,\alpha_n$ in $[0,2\pi)$.
The calculation also needs us to set some number *K*, and the original paper recommends *K*=10.

1. We need to calculate a mean value $\overline{b_j}$ for all *j* values from 1 to 2*K*.

$$\overline{b_j} = \frac{1}{n}\sum_{i=1}^{n} b_j(\alpha_i)$$

Where

$$b_j(x) = \begin{cases} \sqrt{2}sin(0.5jx), & if\ j\ is\ even \\ \sqrt{2}cos(0.5(j+1)x), & if\ j\ is\ odd \end{cases}$$

2. We can then calculate a value N_{2k} for every *k* value from 1 to *K*, as follows

$$N_{2k} = n\sum_{j=1}^{2k}\left(\overline{b_j}\right)^2$$

3. We can then calculate a value *L* for each value of *k* in 1 to *K*.

$$L(k) = N_{2k} - 2klog_e(n)$$

4. We then find *S* as the value of *k* that maximises *L*(*k*)

The final test statistic is the N_{2S}.
The associated p-value has to be evaluated by simulation. A high value of the test statistic suggests significance, and so the p-value is the fraction of samples from a uniform distribution which generate equal or larger values than the test sample.

Hermans & Rasson (1985) offer a family of tests controlled by the value of a parameter β. For the simplest case where β tends to infinity, the test statistic *T* is given by

$$T = \frac{1}{n}\sum_{i=1}^{n}\sum_{j=1}^{n}\left|sin(\alpha_i - \alpha_j)\right|$$

Again we find the p-value by simulation. However, note that a small T value suggests significance, and so the p-value is the fraction of samples from a uniform distribution which generate smaller T values than the test sample.
However, the authors caution that this choice has particularly poor power against unimodal deviations, and instead recommend β= 2, which changes the test statistic to

$$T = \frac{1}{n}\sum_{i=1}^{n}\sum_{j=1}^{n}\pi - \left|\pi - \left|\alpha_i - \alpha_j\right|\right| + 2.895\left|sin(\alpha_i - \alpha_j)\right|$$
