# Supplementary material for: Circular data in biology: advice for effectively implementing statistical procedures
Source: Behav Ecol Sociobiol. 2018 Jul 11;72(8):128. doi: 10.1007/s00265-018-2538-y (PMC6060829; doi:10.1007/s00265-018-2538-y)
Supplement: Supplementary file 3 — (PDF 109 kb) [file 265_2018_2538_MOESM3_ESM.pdf]

Online Resource 3: R code to implement the Hermans-Rasson and Bogdan tests.

Behavioural Ecology and Sociobiology

Circular data in biology: Advice for effectively implementing statistical procedures

Lukas Landler[1], Graeme D. Ruxton[2], E. Pascal Malkemper[1,3]

Affiliations

1 Research Institute of Molecular Pathology (IMP), Vienna Biocenter (VBC), Austria

2 School of Biology, University of St Andrews, St Andrews KY16 9TH, UK

3 Department of General Zoology, Faculty of Biology, University of Duisburg-Essen, 45117 Essen, Germany

Corresponding author email address: pascal.malkemper@imp.ac.at

R libraries used

```
library(circular)
library(NPCirc)
```

Hermans-Rasson (calculating the critical value)

```
HermansRassonTunc <- function(sample){
  n <- length(sample)
  total <- 0
  for (i in 1:n){
    for (j in 1:n){ total <- total + pi - abs(pi - abs(sample[i]-
 sample[j]))
    total <- total + (2.895*abs(sin(sample[i]-sample[j]))))}}
  T <- total/n
  return(T)}

HermansRassonT <- cmpfun(HermansRassonTunc)
```

Hermans-Rasson (calculating the p-value)

```
n <- length(sample)
rand= 1000
Random <- sapply(1:rand, function(x)rcircularuniform(n,
 control.circular=list(units="radians")))

RandomT <-as.vector(apply(Random, 2,HermansRassonT))

HRT <- HermansRassonT(sample)
HRTpvalue <- sum(1*(RandomT < HRT)/rand)
```

Bogdan (calculating the critical value)

```
BogdanNunc <- function(sample){
  K <- 10
  n <- length(sample)
  bjbar <- rep(0,(2*K))
  for(j in 1:(2*K)){
    if((j %% 2) == 0) {for (i in 1:n){bjbar[j] <-
 bjbar[j]+(sqrt(2)*sin(0.5*j*sample[i]))}}
    else {for (i in 1:n){bjbar[j] <-
 bjbar[j]+(sqrt(2)*cos(0.5*(j+1)*sample[i]))}}}
  bjbar <- bjbar/n

  N2k = rep(0,K)
  for(k in 1:K){

    for (j in 1:2*k){N2k[k] <- N2k[k]+(bjbar[j]*bjbar[j])}}
  N2k <- N2k*n

  L <- rep(0,K)
  for (k in 1:K){L[k] = N2k[k] - (2*k*log(n))}

  S <- which.max(L)
```

```
  N <- N2k[S]
  return(N)}

BogdanN <- cmpfun(BogdanNunc)
```

Bogdan (calculating the p-value)

```
n <- length(sample)
rand= 1000
Random <- sapply(1:rand, function(x)rcircularuniform(n,
 control.circular=list(units="radians")))

RandomN <-as.vector(apply(Random, 2,BogdanN))

BogN <- BogdanN(sample)
BogNpvalue <- sum(1*(RandomN >= BogN))/rand)
```
